# Seaweeds and Their Natural Products for Preventing Cardiovascular Associated Dysfunction

**DOI:** 10.3390/md19090507

**Published:** 2021-09-07

**Authors:** Bomi Ryu, Young-Sang Kim, You-Jin Jeon

**Affiliations:** 1 Department of Marine Life Science, Jeju National University, Jeju 63243, Korea; 2 Marine Science Institute, Jeju National University, Jeju 63333, Korea; 3 Healthy Seafood Research Center, Jeju National University, Jeju 63243, Korea

**Keywords:** cardiovascular disease, dyslipidemia, hypertension, vascular endothelial cell, seaweed, natural product

## Abstract

Cardiovascular disease (CVD), which involves the onset and exacerbation of various conditions including dyslipidemia, activation of the renin–angiotensin system, vascular endothelial cell damage, and oxidative stress, is a leading cause of high mortality rates and accounts for one-third of deaths worldwide. Accordingly, as dietary changes in daily life are thought to greatly reduce the prevalence of CVD, numerous studies have been conducted to examine the potential use of foods and their bioactive components for preventing and treating CVD. In particular, seaweeds contain unique bioactive metabolites that are not found in terrestrial plants because of the harsh environment in which they survive, leading to in vitro and in vivo studies of their prevention and treatment effects. This review summarizes studies that focused on the beneficial effects of seaweeds and their natural products targeting markers involved in a cascade of mechanisms related to CVD pathogenesis. The purpose of this review is to describe the potential of seaweeds and their natural products for preventing and treating CVD based on in vivo and in vitro studies. This review provides a basis for future research in the field of marine drugs.

## 1. Introduction

According to the National Health and Nutrition Examination Survey, from 2015 to 2018, the prevalence of cardiovascular disease (CVD) including coronary heart disease (CHD), heart failure (HF), and hypertension in adults over 20 years of age is 49.2% (126.9 million people in 2018), whereas the CVD prevalence excluding hypertension (CHD, HF, and stroke only) is 9.3% (26.1 million in 2018) [1]. In another study, the overall prevalence of lipitension, hypertension alone, and hypercholesterolemia alone was found to be 30%, 47%, and 18%, respectively [2]. CVD is the leading cause of global mortality and a major contributor to disability, representing 17.8 million deaths, which accounts for 32% of all global deaths according to the statistics update in 2019 from the World Health Organization [3]. A large proportion of CVD cases is directly related to dietary risks, high systolic blood pressure (BP), high body mass index, high total cholesterol level, high fasting plasma glucose level, tobacco smoking, and low levels of physical activity [3].

Particularly, several medical studies across diverse hospitals and patient populations have revealed that patients with coronavirus disease 2019 (COVID-19) and underlying CVD are at an increased risk for developing severe symptoms, poor prognosis, and high mortality rates [4,5,6]. Although the relationship between COVID-19 and CVD remains unclear, approximately 30–35% of COVID-related deaths are known to be associated with underlying CVD, supporting the close relationship between these conditions [5,7]. Severe acute respiratory syndrome coronavirus 2 (SARS-CoV-2), the etiologic agent of COVID-19, targets the angiotensin-converting enzyme 2 (ACE2) receptor on the host cell receptor by recognizing the viral spike protein; after entering the cells, the virus can cause infections in the heart, vascular tissues, and circulating cells [8,9]. As such, there is growing concern that CVD appears to increase the risk of serious symptoms of COVID-19, particularly in older patients and those with impaired immune system function.

CVD is a class of diseases that occur in the heart and blood vessels (veins and arteries), including heart disease, other vascular diseases, and cerebrovascular diseases [10]. Heart diseases include ischemic heart disease due to the progression of arteriosclerosis. Hypertension, heart failure, arrhythmia, cardiomyopathy, and endocarditis are forms of ischemic heart disease [11]. Vascular diseases include stroke and peripheral vascular diseases [10,11]. Hypertension and dyslipidemia, as the two main risk factors for CVD, typically cause arteriosclerosis by blocking or narrowing the coronary arteries that supply blood to the heart. In particular, plaque buildup leads to artery narrowing, making it more difficult for blood to pass through, which can block blood flow when clots form [12]. In addition, people with high blood pressure are more likely to develop coronary artery disease because high blood pressure exerts an added force against the artery walls. Over time, excess pressure can damage the arteries, making them more vulnerable to narrowing and plaque buildup associated with atherosclerosis. As the narrowed arteries reduce oxygen supply, the hardened surface of the arteries promotes the formation of small blood clots, potentially leading to a heart attack or stroke. Various epidemiological studies have shown that the prevalence of coexisting hypertension and dyslipidemia is 15–31% [12,13]. The coexistence of these two risk factors has more than an additive adverse impact on the vascular endothelium, increasing the risk of atherosclerosis and leading to CVD [14].

Dietary changes in daily life are a major approach used to reduce the prevalence of chronic diseases, such as CVD [14,15]. Accordingly, various studies have begun to reveal that foods and their physiologically active components can affect CVD [14,16]. In this context, marine seaweeds have vast biodiversity because they are exposed to a wide range of environmental factors that differ from those of terrestrial plants, leading to the pro-duction of secondary metabolites with various characteristics and applicability. Various in vitro, in vivo, and clinical studies have reported on the efficacy of seaweeds and their natural products for reducing the risk of CVD [17,18]. For example, several studies have revealed an association between dietary intake of seaweed and increased life expectancy or reduced incidence of certain diseases, such as CVD [19]. The purpose of this review is to describe the potential of using natural products derived from seaweeds to prevent and treat CVD based on in vivo and in vitro studies. In addition, we discuss the mechanisms that may be involved in the beneficial effects of seaweed-derived natural products on CVD.

## 2. Marine Natural Product on Hyperlipidemia

With changes in lifestyle and improvements in living standards, consumption of a high-fat diet has become common, gradually increasing the prevalence of hyperlipidemia. Hyperlipidemia is typically caused by increases in serum total cholesterol (TC), triglyceride (TG), and low-density lipoprotein cholesterol (LDL-C) levels and decreased levels of high-density lipoprotein cholesterol (HDL-C). This condition is reported to be closely correlated with atherosclerosis and is a common cause of CVD [8,20]. Adults in their 40s and 50s with hyperlipidemia have an increased risk of coronary heart disease, including those with a low cardiovascular risk [21]. In addition, it has been reported that long-term use of lipid-lowering agents improves the survival of patients with coronary heart disease to improve patient prognosis [22].

As controlling blood lipid levels is important for preventing and improving CVD, many studies are being conducted to identify active components with lipid-lowering activities. Specifically, the prevalence of obesity-related diseases in people who consume marine products was shown to be low, suggesting that marine products and their active components have lipid-lowering effects [17,23]. Based on this information, various marine products, including seaweed with lipid-lowering effects, have been evaluated to promote the development and utilization of related bioactive components. Table 1 shows the lipid-lowering effects of seaweed extracts observed in in vivo models.

Supplementing the diets of hypercholesterolemic Wistar rats with 21% or 23% of *Himanthalia elongata* or *Gigartina pistillata*, which is equivalent to 8% dietary fiber for four weeks, improved the serum lipid profile as compared to in hypercholesterolemic Wistar rats without dietary intervention [24]. The *Himanthalia* diet significantly reduced the TG content by 28% and increased the HDL-C content by 20%. The diet containing *Gigartina* improved the lipid profile by decreasing TG, TC, or LDL-C levels by 30%, 18%, and 16%, respectively. Villanueva et al. also found that seaweed intake improved the lipid profile [24]. Kumar et al. reported that intake of *Derbesia tenuissima* for eight weeks decreased plasma TG and TC levels by 38% and 17%, respectively, in rats fed a high-fat diet because of the insoluble fiber content (23.4%) [25]. Chan et al. also found that *Gracilaria changii*, which has a high dietary fiber content of 61.29%, significantly improved the lipid profile of high-cholesterol/high-fat Sprague Dawley rats [26]. Rats fed a HF diet supplemented with 5% or 10% *G. changii* exhibited significantly reduced plasma TC, LDL-C, and TG contents. In addition, changes in the lipid profile were observed even in rats given normal feed supplemented with *G. changii* during the experimental period, but the authors explained that the lipid changes were caused by the normal growth process of the experimental model and were not related to the feed supplement. However, a change in the lipid profile of a normal animal model following seaweed intake was reported by Kim et al. [27] and Ruqqia et al. [29]. Kim et al. observed that *Ecklonia cava* had lipid-lowering effects in both normal mice and streptozotocin-diabetic mice, demonstrating the potential of this supplement to prevent the progression of coronary heart disease. Jung et al. [28] further evaluated the properties of phlorotannins from *Ecklonia stolonifera* in vitro. In addition, various seaweeds, including *Rhizoclonium implexum*, *Dictyota indica*, *Padina pavonia*, *Stoechospermum marginatum*, *Stokeyia indica*, *Jolyna laminarioides*, *Sargassum binderi*, and *Melanothamnus afaqhusainii* showed lipid-lowering effects by reducing TC, TC and LDL-C and increasing HDL-C in normal rats according to Ruqqia et al. [28]. The authors emphasized the medical importance of seaweed, as consumption of seaweed not only inhibited the progression of CVD, but also regulated the accumulation of lipids in daily life and may play an important role in improving the survival of humans. Based on the lipid-lowering effect in normal rats, Ruqqia et al. further investigated *J. laminarioides*, *S. binderi*, and *M. afaqhusainii* for their antihyperlipidemic effects in Triton-induced hyperlipidemic rats and in high-fat diet-induced hyperlipidemic rats. They found that the brown seaweeds *J. laminarioides* and *S. binderi* significantly decreased TG levels by 31.6% and 33% in high-fat diet-induced hyperlipidemic rats. Jimenez-Escrig and Sanchez-Muniz reported that alginic acid and alginic acid isolated from brown algae play important roles in lowering blood cholesterol levels in rats by decreasing intestinal cholesterol absorption [38]. Patil et al. noted that sulfated polysaccharides in brown algae delay the intestinal absorption of cholesterol or promote cholesterol excretion [39]. Cuong et al. produced fucoidan, a sulfated polysaccharide from the brown seaweed *S. henslowianum*, and found that it lowered cholesterol, TG, and LDL-C levels when administered orally at 100 mg/kgP/day to obese rats [30]. The red seaweed *M. afaqhusainii*, which contains 0.46 ± 0.01% sterols, also exerted lipid-lowering effects. In the 1970s, Bhakuni and Silva reported that cholesterol is the most commonly occurring sterol in red seaweed and can reduce blood cholesterol levels [40]. In addition, Ruqqia et al. found that the non-toxic sterols of red algae can lower blood cholesterol and fat accumulation in the heart and liver [29]. In a clinical study, carrageenans from red seaweed significantly decreased cholesterol levels (16.5%) and LDL-C levels (33.5%), leading to a reduced atherosclerotic index [31]. Dousip et al. compared the cholesterol-lowering properties of the red seaweed *Kappaphycus alvarezii* and brown seaweed *Sargassum polycystum* [32]. *Kappaphycus alvarezii* contains 42.09 ± 0.97% carrageenan and *S. polycystum* contains 8.98 ± 0.33% alginate. *Sargassum polycystum* consumption significantly decreased the plasma cholesterol level by 37.52% over an eight-week treatment period compared to *K. alvarezii*. Jiménez-Escrig and Sánchez-Muniz reported that the antihyperlipidemic activity of alginate in brown algae is affected by the degree of polymerization [38]. Accordingly, Dousip et al. explained that the cholesterol-lowering effect was lowered as the alginate of *S. polycystum* contained a high-molecular weight alginate polymer [32]. In addition, the beneficial effects of polysaccharides and ulvans in green seaweed extracted from *Ulva fasciata*, *Ulva lactuca*, and *Monostroma nitidum* were suggested to improve lipid profiles [33,34,35]. Marine-derived active components such as fucoidan and fucoxanthin have also been evaluated and shown to have beneficial effects on lipid profiles in in vivo models [36,37].

## 3. Marine Natural Products Affect Endothelial Dysfunction

Atherosclerosis, mainly caused by hypertension and dyslipidemia, begins with dysfunction of vascular endothelial cells and develops into CVD via plaque accumulation and related lesion formation in the blood vessels [12].

**Figure 1:**
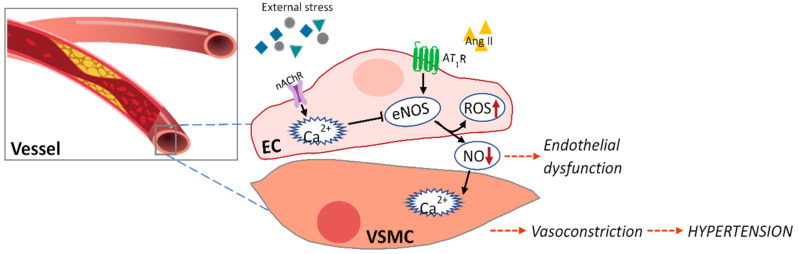
Crosstalk between endothelium (EC) and vascular smooth muscle cells (VSMCs) in hypertension.

Vascular endothelial dysfunction is caused by (1) decreased eNOS activation by reduced intracellular Ca^2+^ level in the endothelium, (2) decreased bioavailability of nitric oxide produced from eNOS, (3) increased production of endothelial-derived vasoconstrictor factors, and (4) increased levels of oxidative stress and inflammation-inducing cytokines [41] (Figure 1). Table 2 shows the effects of various seaweed components on endothelial dysfunction in in vitro and in vivo models.

Alam et al. reported that the natural carotenoid astaxanthin extracted from microalgae *Haematococcus pluvialis* can penetrate the endothelial cell membrane and significantly inhibit ROS, thereby inhibiting oxidative stress in ISO-induced myocardial infarction and cardiac hypertrophy in rats, suggesting its cardioprotective action [42]. Zhao et al. found that astaxanthin protects against endothelial dysfunction of the aorta in diabetic rats and predicted the molecular mechanism involved in their effects [43]. They suggested that astaxanthin can attenuate blunted endothelium-dependent vasodilator responses to acetylcholine, upregulate endothelial nitric oxide synthase expression, and decrease excessive oxidative stress and endothelial dysfunction. Lee et al. isolated dieckol from the brown seaweed *E. cava* and found that it protected human umbilical vein endothelial cells damaged by high glucose via its antioxidant properties [44]. In addition, the positive effects of eckol and its derivatives, including dieckol from the brown seaweed *Ecklonia bicyclis*, were investigated in both human umbilical vein endothelial cells and mice [45]. They suggested that the abundance of hydroxyl groups of eckol and its derivatives contribute to their vascular barrier protective functions. Another phlorotannin, diphlorethohydroxycarmalol (DPHC) isolated from *Ishige okamurae*, was observed to have vasodilatory effects by increasing nitric oxide production and Ca^2+^ release in endothelial cells via stimulating the Ach receptor and VEGF-receptor 2 [46]. The author further demonstrated the vasodilatory ability of DPHC in Tg(flk:EGFP) transgenic zebrafish. In addition, another crucial components in brown seaweed, the sulfated polysaccharides extracted from *Padina tetrastromatica*, were investigated for their effect on ISO-induced myocardial infarction in a rat model [47]. ISO-induced hyperlipidemia, endothelial dysfunction, and inflammatory reactions were significantly reduced by the sulfated polysaccharides. Specifically, the authors emphasized that sulfated polysaccharides can be used as a new functional food ingredient for CVD, as they showed therapeutic ability similar to that of aspirin, a reference drug.

## 4. Marine Natural Products Inhibit ACE

**Figure 2:**
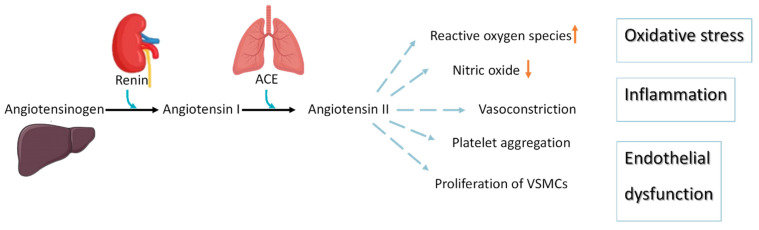
The renin–angiotensin system is responsible for cardiovascular disease progression.

Hypertension occurs in 25–30% of the population in developed countries and is used as a biomarker for increased cardiovascular risk [1]. Blood pressure monitoring is the most commonly used procedure for diagnosing hypertension. In normal individuals, the systolic and diastolic blood pressure values are less than 120 and 80 mmHg, respectively. The renin–angiotensin–aldosterone system regulates blood pressure [48]. Angiotensinogen is produced by the liver and circulates continuously in the plasma. Renin produced by the kidneys is responsible for cleaving angiotensinogen into angiotensin I in response to reduced blood flow. The produced angiotensin 1 (ACE-1) is cleaved by angiotensin-converting enzyme (ACE), which in turn produces angiotensin II (ACE-2) [48]. In addition, ACE-2 is typically associated with vasoconstriction and hypertension by modulating blood pressure and is involved in hyperplasia and hypertrophy of vascular smooth muscle cells in CVD progression (Figure 2). Thus, inhibition of ACE-I is a well-established approach for treating hypertension, and many authors have used seaweed extract and its components to screen for components in seaweed that inhibit this enzyme (Table 3).

Cha et al. investigated the ACE-I inhibitory activities of red seaweeds (26 species) and found that *Lomentaria catenate* and *Lithophyllum okamurae* exhibited the strongest ACE inhibitory activity with IC_50_ (μg/mL) values of 13.78 and 12.21, respectively, indicating their potential as ACE-I inhibitors [49]. In addition, the extracts of *E. cava* and their phlorotannin polyphenol compounds phloroglucinol, triphlorethol-A, phlorofucofuroeckol A, dieckol, eckol, eckstolonol, and 6,6-bieckol, which were isolated from the brown seaweed *Ecklonia* sp., were compared for their ACE inhibitory activity [50,51,52]. The authors suggested that *E. cava* is a promising agent for ACE-1 inhibition and that its phlorotannin can contribute to these inhibitory effects. Additionally, Ko et al. demonstrated that 6,6-bieckol, which has been shown to inhibit ACE, contributes to the stabilization of the ACE active site. The author analyzed the antihypertensive efficacy of 6,6-bieckol in a hypertensive rat model by measuring the systolic blood pressure and analyzing the binding affinity of each phlorotannin to the active site of the targeting ACE-1 protein [52]. As a result, they suggested that 6,6’-bieckol induced nitric oxide in vascular endothelial cells and effectively reduced systolic blood pressure in the spontaneous hypertension model, suggesting that this agent can be used to treat hypertension. Other studies conducted by Vijayan et al. [53] and Nagappan et al. [54] reported the beneficial effects of polyphenols in seaweed by *Sargassum wightii* and *Sargassum siliquosum* on inhibiting ACE-1. Another study analyzed the potential of total phenolic and carbohydrate contents of seaweeds, *Lessonia nigrescens*, *Macrocystis pyrifera*, and *Durvillaea antarctica* as ACE-1 inhibitors [55]. The authors reported that the total phenolic and carbohydrate contents in these brown seaweeds after enzymatic extraction compared to the conventional method (maceration) may be responsible for their superior ACE inhibitory activity. Zhu et al. reported that D-polymannuronic sulfate, a type of sulfated polysaccharide obtained from brown alga, significantly reduced systolic blood pressure and diastolic blood pressure by increasing the serum nitric oxide content in hypertensive animals and significantly lowering the plasma concentrations of Ang II and endothelin-1 [56]. In addition, sulfated polygalactans from the red seaweeds *K. alvarezii* and *Gracilaria opuntia* were shown to have ACE-1 inhibitory activity, demonstrating their potential as therapeutic candidates for preventing hypertensive disorders [57].

## 5. Marine Natural Products Affect ACE2 for SARS-CoV-2 Entry into Cells

As COVID-related mortality rates are high in patients with underlying CVD, many studies are underway to find new targeted drugs to prevent the uncontrolled spread of SARS-CoV-2 [5,7]. Recent studies have suggested that SARS-CoV-2 uses ACE2, a component of the renin–angiotensin-aldosterone system, as an entry receptor in cells [58]. Accordingly, many studies are being conducted to identify new target drugs to inhibit the ACE2-binding affinity of the SARS-CoV-2 spike. Table 4 lists reports of components derived from seaweeds that can modulate ACE2 to prevent SARS-CoV-2 infection.

Syahputra et al. screened secondary metabolites against SARS-CoV-2 isolated from 36 marine organisms with high biodiversity from Indonesia [59]. Molecular docking was used to screen the potential of each drug component for targeting a protein molecule. They suggested that inhibiting the enzymatic activity of ACE2 could prevent spike proteins from binding to ACE2. Accordingly, among the marine derived natural products dieckol, phlorofucofuroeckol A, fucoidan, thalassodendrone, and thalassiolin D were selected as competitors and protective agents against SARS-CoV-2 infection, as they form stable complexes with ACE2. In addition, Salih et al. reported that sulfated polysaccharides from brown seaweeds evaluated by molecular docking and dynamic simulation experiments revealed potential interactions with the spike protein of SARS-CoV-2, and the receptor binding domain of ACE2 was suggested as a potential target of SARS-CoV-2 inhibitors [60]. Youssef described the potential of alkaloids derived from the genus *Aspergillus*, a marine-derived fungi, based on the structural stability of ACE2 bound to alkaloids in an in silico study [61]. They reported that fumigatoside E and aspergicin as introduced alkaloids were the most suitable active sites for ACE2, and they predicted a positive effect in preventing and eradicating SARS-CoV-2 infection that should be further studied.

## 6. Conclusions

Seaweed is a valuable source of compounds with various uses, including improving cardiovascular health. In this review, the prevention of CVD by consuming seaweed was described, and the effects of seaweed and its components on the pathogenesis of CVD were evaluated. In addition, various studies including in vitro, in vivo, and related clinical evidence have shown that the onset and progression of CVD were improved through dietary supplementation with seaweed. However, to use seaweed and related ingredients as medical or functional foods, additional studies of their effects on the mechanism of CVD and their clinical relevance are required. These studies can be utilized to develop functional foods and pharmaceuticals for preventing and improving CVD.

## Figures and Tables

**Table 1:** Lipid-lowering effect of seaweed and its components in in vitro and in vivo models.

Seaweeds	Experimental Models	Effects (% or mmol/L)	Ref.
*Himanthalia elongate*, B	Hypercholesterolaemic wistar rats : 21% in diets for four weeks	↓: TG by 28% ↑: HDL-C by 20%	[24]
*Gigartina pistillata*, R	Hypercholesterolaemic wistar rats : 23% in diets for four weeks	↓: TG by 30%, TC by 18%, LDL-C by 16%	[24]
*Derbesia tenuissima*, G	High-Fat Fed Rats : 5% in diets for eight weeks	↓: TG by 38% and TC by 17%	[25]
*Gracilaria changii*, R	High-cholesterol/high-fat Sprague Dawley rats : 5% or 10% in diets for eight weeks	5% ↓: TC by 39.19%, LDL-C by 36.36%, TG by 25.45% 10% ↓: TC by 40.34%, LDL-C by 35.95%, TG by 30.91%	[26]
*Ecklonia cava*, B	STZ-diabetic mice : 5% in diets for four weeks	↓: TG by 72%, TC by 53%, and LDL-C by 78%	[27]
*Ecklonia stolonifera,* B	3T3-L1 preadipocyte cells : Phloroglucinol, Eckol, Dieckol, Dioxinodehydroeckol, Phlorofucofuroeckol A, 12.5 to 100 µM, eight days	↓: lipid accumulation. ↓: level of adipocyte marker genes	[28]
*Rhizoclonium implexum*, G	A: Adult Albino rats (Sprauge-Dawley) T: Triton-induced hyperlipidaemic rats H: High-fat diet-induced hyperlipidaemic rats : 10 mg/200 g/day for 12 days, OA	A: ↓: TC by 14.4%, TG by 26.4%, LDL-C by 25.5% ↑: HDL-C by 3.1%	[29]
*Dictyota Indica*, B		A:↓: TC by 13.5%, TG by 24.6%, LDL-C by 25.4% ↑: HDL-C by 3.1%	
*Padina pavonia,* B		A: ↓: TC by 26.5%, TG by 37%, LDL-C by 54.3% ↑: HDL-C by 23.5%	
*Stoechospermum marginatum,* B		A: ↓: TC by 21.7%, TG by 40.2%, LDL-C by 30% ↑: HDL-C by 6.2%	
*Stokeyia indica,* B		A: ↓: TC by 22.6%, TG by 17.2%, LDL-C by 40.9% ↑: HDL-C by 0.7%	
*Jolyna laminarioides*, B		A: ↓: TC by 10%, TG by 49%, LDL-C by 28.7% ↑: HDL-C by 23.5%	
		T: ↓: TC by 41.2%, TG by 25.2%, LDL-C by 92.4% ↑: HDL-C by 60.6%	
		H: ↓: TC by 19.8%, TG by 31.6%, LDL-C by 34.5% ↑: HDL-C by 33.1%	
*Sargassum binderi*, B		A: ↓: TC by 20.5%, TG by 4.2%, LDL-C by 28.0%, HDL-C by 17.4%	
		T: ↓: TC by 37.6%, TG by 52.2%, LDL-C by 51.1% ↑: HDL-C by 8.6%	
		H: ↓: TC by 2.5%, TG by 33%, LDL-C by 2.9% ↑: HDL-C by 30%	
*Melanothamnus afaqhusainii*, R		A: ↓: TC by 10.3%, TG by 36.1%, LDL-C by 17.5% ↑: HDL-C by 5%	
		T: ↓: TC by 35.2%, TG by 43.2%, LDL-C by 71.4% ↑: HDL-C by 57.3%	
		H: ↓: TC by 14.2%, TG by 25.1%, LDL-C by 5.4% ↑: HDL-C by 16.8%	
*Fucoidan from Sargassum henslowianum* (B)	High-fat diet albino mice of BALB/c strain : 100 mg/kg/day for four weeks, OA	↓: TC by 21.09%, TG by 6.35%, LDL-C by 18.74%	[30]
*Carrageenans*	Ischemic Heart Disease (IHD) patients : 250 mg/day for 20 days, OA	↓: TC by 16.5%, LDL-C by 33.5%	[31]
*Kappaphycus alvarezii*, R	High-cholesterol diet Male Sprague–Dawley rats : 300 mg/kg/day for eight weeks, OA	↓: TC by 1.91±0.62%, TG by 0.65±0.05, LDL-C by 1.65±0.08 (mmol/L) ↑: HDL-C by 1.74±0.08 (mmol/L)	[32]
*Sargassum polycystum*, B		↓: TC by 1.91±0.62%, TG by 0.65±0.05, LDL-C by 1.65±0.08 (mmol/L) ↑: HDL-C by 1.74±0.08 (mmol/L)	
*Ulva fasciata*, G	High-cholesterol diet rats : 175 mg/kg/day for four weeks, OA	↓: TC by 46.43%, TG by 69.03%, LDL-C by 81.04% ↑: HDL-C by 668.31%	[33]
*Ulva lactuca*, G	Hypercholesterolemic diet rats : 250 mg/kg/day for four weeks, OA	↑: HDL-C by 180%	[34]
*Monostroma nitidum*, G	lipid-loaded hepatocytes (HepG2 cell line) : 200 µg/mL for one day	↓: Cellular cholesterol by 36%, TG by 31%,	[35]
Fucoidan	Hyperlipidemic diet mice : 10 to 50 mg/kg/day for four weeks, OA	↓: TC, TG and LDL-C ↑: HDL-C	[36]
Fucoxanthin	Hyperlipidemic diet mice : 21% in diets for six weeks, OA	↓: Liver TG synthesis, adipocyte fatty acid synthesis, and cholesterol-regulating enzyme activity ↑: Plasma HDL-C ↑: Fecal TG level	[37]

B: brown seaweed; R: red seaweed; G: green seaweed; STZ: streptozotocin; OA: oral administration.

**Table 2:** Effects of seaweed components on endothelial dysfunction.

Component	Experimental Model	Effects	Ref
Astaxanthin	ISO-induced myocardial infarction and cardiac hypertrophy model in rats : 25 mg/kg/day for two weeks, OA	↓: ROS generation in heart tissue ↓: Oxidative damage ↑: Antioxidant enzyme activity	[42]
	STZ-induced diabetes in male rats : 10 mg/kg/d, OA	↓: Blunted endothelium-dependent vasodilator responses to Ach ↓: Aorta-induced oxidative stress and LOX-1 levels ↑: eNOS levels	[43]
Dieckol	High glucose stimulation in cultured vascular endothelial cells. : 10 or 50 μg/mL	↓: ROS production ↓: iNOS, COX-2, and NF-κB levels	[44]
Eckol and its derivates	Cultured vascular endothelial cells/mice : 50∼200 μg/mL	Protects the vascular barrier	[45]
DPHC from *Ishige okamurae*	Cultured vascular endothelial (EA.hy926) cells)/Tg(flk:EGFP) Transgenic Zebrafish : 100 μM/0.6 μM	↑: Ach receptor and VEGF receptor 2 ↑: NO production ↑: Ca^2+^ release ↑: Endothelium vasodilation	[46]
Sulfated polysaccharides from *Padina tetrastromatica*	ISO induced myocardial infarction in rats : 50 mg/kg/day for 12 days, OA	↓: hyperlipidemia ↓: Endothelial dysfunction ↓: Inflammatory reactions	[47]

ISO: isoproterenol; OA: oral administration; ROS: reactive oxygen species; STZ: streptozotocin; Ach: acetylcholine; LOX-1: lectin-like oxidized low-density lipoprotein receptor-1; eNOS: endothelial nitric oxide synthase; iNOS: inducible nitric oxide synthase; COX-2: cyclooxygenase-2; NF-κB: nuclear Factor kappa B; DPHC: diphlorethohydroxycarmalol; VEGF: vascular endothelial growth factor; NO: nitric oxide.

**Table 3:** Effects of seaweed extract and its components on ACE-Ⅰ inhibition.

Seaweed Species	Extraction/Compound	Inhibition	Ref
Twenty-six red algae	Aqueous extract at 20 °C	IC_50_ (μg/mL) = *Lomentaria catenate*, 13.78; *Lithophyllum okamurae*, 12.21; (* IC_50_ of captopril is 0.05 ± 0.8 μg/mL)	[49]
*Ecklonia* sp, B	Ethanol extract of *Ecklonia* *cava*, Phloroglucinol, Triphlorethol-A, Phlorofucofuroeckol A, Dieckol, Eckol, Eckstolonol, and 6,6′-bieckol	IC_50_ = *Ecklonia* *cava* EtOH extract, 0.96 mg/mL Phloroglucinol, 2.57 ± 0.09 Triphlorethol-A, 2.01 ± 0.36 Phlorofucofuroeckol A, 12.74 ± 0.15 μM Dieckol, 1.47 ± 0.04 Eckol, 2.01 ± 0.36 Eckstolonol, 2.95 ± 0.28 6,6′-bieckol, 0.42 mM	[50,51,52]
*Sargassum wightii*, B	Ethyl acetate extract (Polyphenol-rich extract (49.10 ± 2.52 µg/mg)	IC_50_ = 56.96 µg/mL	[53]
*Sargassum siliquosum*, B	MeOH extract (Fucoxanthin-rich fractions)	IC_50_ = 0.94 ± 0.67 mg/mL	[54]
*Lessonia nigrescens*, B	Maceration	IC_50_ = 48.57 ± 8.04 μg/mg	[55]
	Cellulase	IC_50_ = 19.71 ± 2.04 μg/mg	
	α-Amylase	IC_50_ = 10.10 ± 1.55 μg/mg	
*Macrocystis pyrifera*, B	Cellulase	IC_50_ = 82.87 ± 8.82 μg/mg	
	α-Amylase	IC_50_ = 11.93 ± 0.94 μg/mg	
*Durvillaea antarctica*, B	α-Amylase	IC_50_ = 7.80 ± 0.69 μg/mg	
*Pelvetia canaliculata*, B	D-Polymannuronic sulphate	↑ production of NO; ↓ concentrations of Ang II and ET 1; ↓ Blood pressure;	[56]
*Kappaphycus alvarezii*, R	Sulphated polygalactans	IC_50_ = 0.02 μg/mL	[57]
*Gracilaria opuntia*, R	Sulphated polygalactans	IC_50_ = 0.70 μg/mL	

IC_50_: inhibition concentration at 50%; ACE: angiotensin-converting enzyme.

**Table 4:** Effects of natural products derived from seaweed on anti-infective potential determined in in silico studies of ACE2.

	Binding Free Energy Value (∆G) for hACE-2	Chemical Structure	Ref
Dieckol (*Ecklonia cava*)	−10.23 ^a^	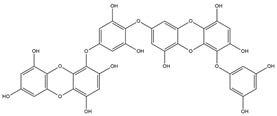	[59]
Phlorofucofuroeckol A (*Ecklonia* sp.)	−9.73 ^a^	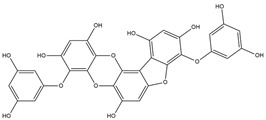	
Fucoidan (Brown algae)	−4.9 ^a^	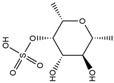	
Thalassodendrone (*Thalassodendrin ciliatum*)	−8.65 ^a^	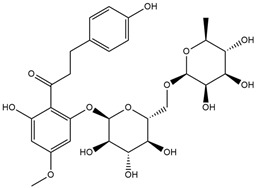	
Thalassiolin D (*Thalassodendrin* sp.)	−8.21 ^a^	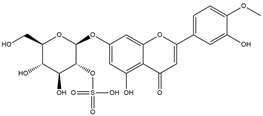	
Sulfated polymannuroguluronate (SPMG)	−5.4 ^b^	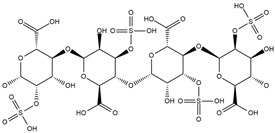	[60]
lambda-carrageenan	−5.0 ^b^	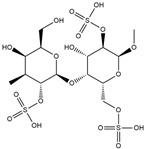	
Heparin	−5.0 ^b^	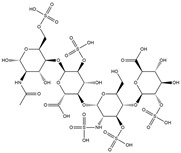	
Fumitremorgin C ^C^	−2.86	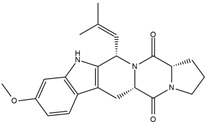	[61]
12,13-Dihydroxy fumitremorgin C ^C^	−2.88	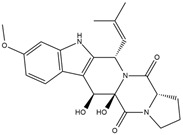	
Fumigatoside E ^C^	−21.17	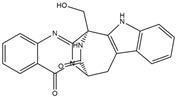	
Fumigatoside F ^C^	−13.81	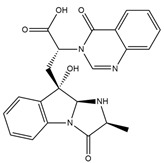	
Versicoloid A ^C^	−1.86	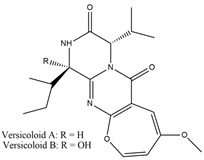	
Versicoloid B ^C^	−2.66		
Aspergicin ^C^	−17.66	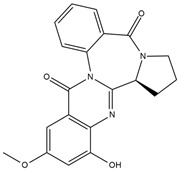	
Stephacidin A ^C^	−0.584	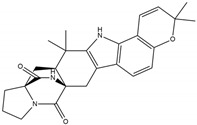	

Note ^a^: human ACE 2 receptor (PDB ID: 1R42); ^b^: human ACE 2 receptor (PDB code: 1R42); ^C^: human ACE 2 receptor (PDB code: 1R4L).
